# Assessing seed characteristics for improved winter survival of late-fall-seeded lentils

**DOI:** 10.3389/fpls.2026.1802566

**Published:** 2026-04-02

**Authors:** Prerana Upretee, Manjula S. Bandara, Randy W. Purves, Yongfeng Ai, Lawrence V. Gusta, Karen K. Tanino

**Affiliations:** 1 Department of Plant Sciences, University of Saskatchewan, Saskatoon, SK, Canada; 2 MCB Agric-Research Consulting, Brooks, AB, Canada; 3 Centre for Veterinary Drug Residues, Canadian Food Inspection Agency, Saskatoon, SK, Canada; 4 Department of Food and Bioproduct Sciences, University of Saskatchewan, Saskatoon, SK, Canada

**Keywords:** freezing tolerance, late fall seeding, LD50 (lethal duration 50%), lentil, LT50(lethal temperature 50%), seed characteristics, water uptake

## Abstract

Late fall seeding (aka dormant seeding) can offer significant benefits over spring seeding, including earlier crop maturity, increased grain yield, and reduced risk of frost damage. However, this practice in Western Canada has not yet been adopted due to failures of canola crop establishment the following spring. A series of experiments was conducted on lentil (*Lens culinaris* Medik.) seeds to identify which characteristics are associated with winter survival and freezing tolerance. The lentil crop was used as a model system due to its variation in seed characteristics. We evaluated 38 genotypes for water uptake at +2 °C and freezing tolerance (LT_50_, LD_50_). Seed characteristics, including thousand-seed weight (TSW), surface area, volume, coat thickness, starch, protein and phenolic content, were quantified to determine their impact on water uptake and freezing tolerance. The total water uptake amount was positively and linearly correlated with TSW, seed surface area, volume and starch content, whereas it was negatively correlated with protein and phenolic content. Seeds that were frozen after imbibition exhibited lower germination percentages than seeds frozen without prior imbibition, indicating increased hydration is a key factor contributing to the loss of freezing tolerance. These findings suggest that seed morphological traits and biochemical composition modulate freezing tolerance primarily through their influence on water uptake dynamics. This research may help improve winter survival of fall-seeded spring crops, such as lentils, enabling a shift from conventional spring planting to late-fall seeding, potentially transforming crop establishment practice on the semi-arid Canadian prairies.

## 1 Introduction

Late-fall seeding can offer several potential benefits to producers, including earlier maturity, yield increases of about 40% and a 5% rise in oil concentration in canola (Kirkland and Johnson, 2000; Johnston et al., 2002). In addition to producing higher yields, canola seeds from fall planting are typically larger and more vigorous than those produced from spring planting (Gusta et al., 2004). However, a key challenge in managing fall-seeded spring crops is preventing stand loss caused by premature germination due to seed water uptake (Johnston et al., 2002). The decline in freezing tolerance during the transition from dry to germinating seeds is closely associated with an increase in seed water content (Hawkins et al., 2003).

Seed moisture content (MC) plays a critical role in freezing survival. Seeds with lower MC are better able to withstand freezing because they are less prone to intracellular ice formation, a major cause of irreversible membrane and structural damage (Murray et al., 1988; Vernon et al., 1999; Jaganathan et al., 2020). In tender tissue such as hydrated seeds, ice formation inside or outside cells causes mechanical injury and dehydration, ultimately leading to cell death.

Seed germination begins with imbibition, the process of water absorption that activates respiratory and enzymatic activity, leading to radicle emergence (Bewley and Black, 2013). Imbibition is a primarily physical process involving water uptake, gas release and temperature change. These processes activate enzymes, convert starches to sugars, and support nutrient transport to the developing embryo (Kranner et al., 2010; Rajjou et al., 2012). The rate of water uptake is pivotal for germination and depends on the seed’s water permeability, which is influenced by factors, such as temperature, seed shape, composition and initial water content (de Souza and Marcos-Filho, 2001; Copeland and McDonald, 2012; Upretee et al., 2024).

Several seed characteristics determine imbibition behavior. Temperature has a strong impact on both the rate of water uptake and the reactivation of metabolism (Weitbrecht et al., 2011; Bewley and Black, 2013). In addition to temperature, factors, such as seed coat biochemistry, seed coat thickness, seed size, surface area, and volume also influence water uptake (Smýkal et al., 2014). Seed coat color has been linked to differences in water absorption among species. White-seeded varieties of French bean (Powell et al., 1986), common bean (Borji et al., 2007), faba bean (Kantar et al., 1996), chickpea (Lamichaney et al., 2017) and rapeseed (Zhang et al., 2008) differ in water uptake and germination compared to respective dark-seeded types. These differences stem from the presence or absence of phenolic compounds such as flavonols, anthocyanidins and condensed tannins (Beninger and Hosfield, 2003). Although there are exceptions, in general, a positive relationship exists between seed coat color and total phenolic content (Elessawy et al., 2020). Higher phenolic and lignin levels in colored seeds contribute to thicker seed coats and slower water uptake (Kannenberg and Allard, 1964). Seed size also influences water absorption through its effect on surface area. Generally, larger seeds absorb water more slowly due to their lower surface area-to-volume ratio (Gürtaş et al., 2001). However, some studies have reported exceptions; for instance, large-seeded chickpea cultivars absorb water faster despite a smaller specific surface area, as compared to smaller seeds, suggesting that other factors also play a role (Hung et al., 1993).

Understanding these seed characteristics is essential for analyzing the water uptake process, as different traits act independently or interactively to influence it. We hypothesized that seed morphological and biochemical traits govern water uptake, determining freezing tolerance and overwinter survival. The objective of this study was to identify seed characteristics linked to water uptake and freezing tolerance, with the expectation that at least one of these traits would significantly influence winter (freezing) survival. The objective of this study was to investigate the effect of seed morphological, biochemical, and anatomical characteristics on water uptake.

## 2 Materials and methods

### 2.1 Seed material

Lentil (*Lens culinaris* Medik.) was considered as a model plant due to its variability in seed traits, such as seed size, seed coat color and cotyledon color. This inherent diversity made lentil suitable for testing hypotheses related to seed characteristics and water uptake. The lentil lines/cultivars were obtained from the Crop Development Centre (CDC) at the University of Saskatchewan and selected based on physical seed traits, such as circularity, diameter, plumpness, height, and 1000-seed weight, along with cotyledon color (red, yellow and green). Among the selected 38 lines, 21 lines had red cotyledons, 15 had yellow and two had green cotyledons (**Supplementary Tables 1**, **2**). The lentil seeds were produced in 2017 in Saskatoon, SK, Canada.

### 2.2 Final quantity of water absorption

#### 2.2.1 At room temperature (23°C)

The water absorption of dry seeds (30 seeds per genotype) was monitored over 24 hours. The water uptake rate was determined using Equation 1, following the method of Chapman et al. (1978) (Chapman et al., 1978). Water absorption capacity was measured using the teabag method described by Buchholz (1998) (Buchholz and Graham, 1998) with three replicates, with each replicate placed in a separate organza mesh bag. The teabag method involves repeated handling of samples at 30-minute intervals, which could potentially introduce minor measurement variability. The seeds were immersed in 250 mL of deionized water at +23 °C for 24 hours. The cups were covered to maintain darkness. Every 30 minutes, the seeds were removed, blotted dry, weighed, and then returned to the water. Weighing continued until constant weight was gained across three consecutive measurements.

Water absorption rate was calculated as follows:

(1) $$W\left(\%\right)=\frac{W_{s}-W_{i}}{W_{i}}*100$$

where W is water absorption rate (%); W_s_ is weight after soaking; W_i_ is initial weight.

#### 2.2.2 At +2 °C

Fourteen lentil lines/cultivars with red, yellow, and green cotyledons and high, medium and low water absorption capacity were selected based on their final quantity of water absorption measured after 24 hours at +23 °C in (a). Water uptake experiments were then conducted at +2 °C, simulating the average soil temperature in late fall in Saskatoon (2000–2015). In these experiments, seeds were immersed in deionized water at a temperature of +2 °C, and weight measurements were recorded at hourly intervals.

#### 2.2.3 Experimental design and statistical analysis

The water absorption of seeds for each crop species was compared in a completely randomized design with three replicates for each treatment using a fixed repeated model, and the significance of treatments (lines/cultivars) was determined at a 5% probability level using RStudio (version 2023.06.2).

### 2.3 Freezing tolerance of imbibed lentil seeds

Three lentil cultivars were selected based on cotyledon color (green, red, and yellow) and the quantity of water uptake over time at +2 °C. For each cotyledon color, the cultivar with the highest water absorption was chosen. Although each represented the maximum absorber within its respective color group, the three cultivars differed significantly.

#### 2.3.1 Determination of the LT_50_ of imbibed seeds

**Figure 1:**
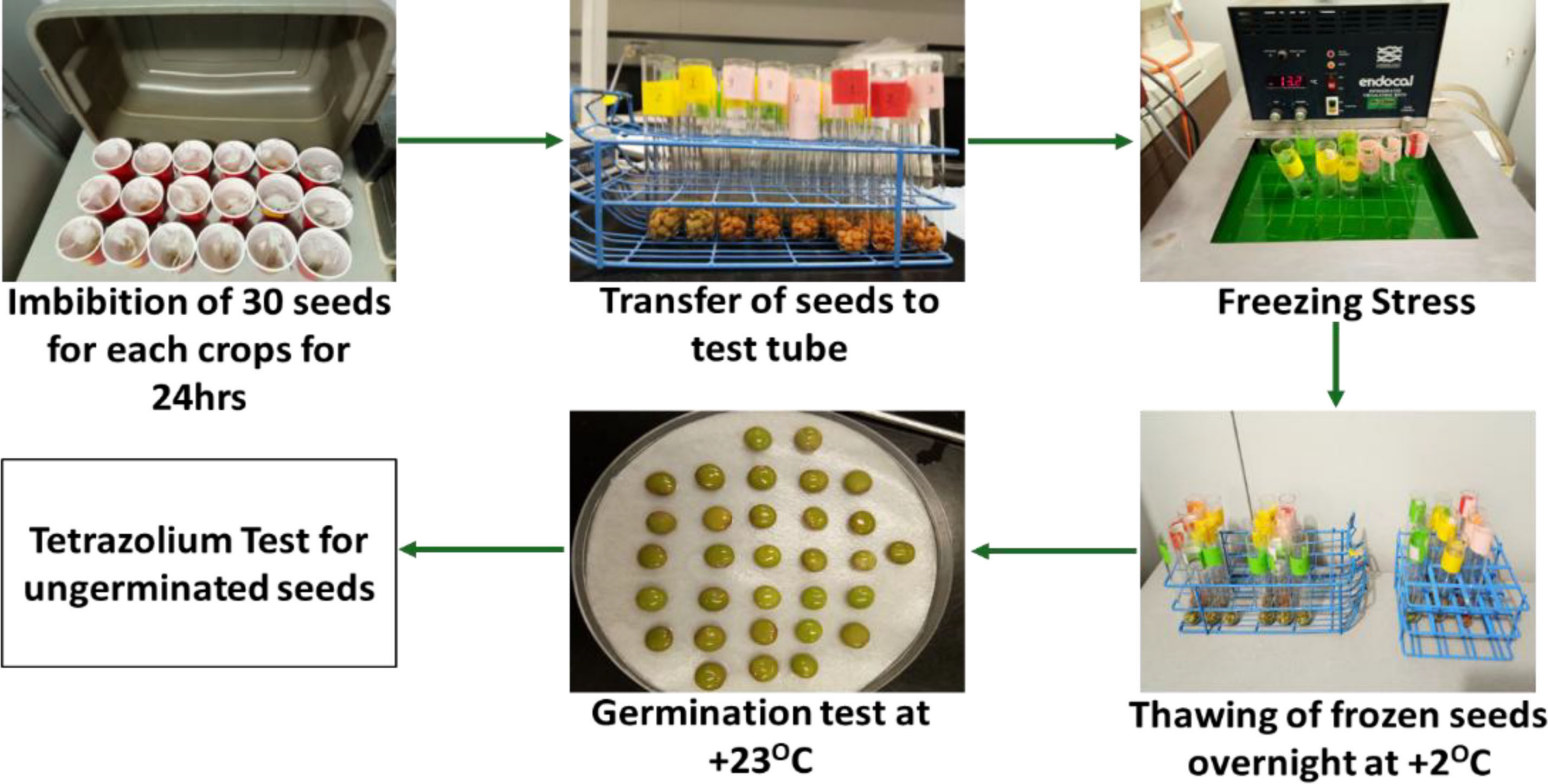
Steps involved in the LT_50_ freezing test. Seeds were nucleated with shaved ice at -2 °C and the temperature was subsequently lowered at the rate of 2 °C for one hour. Samples were removed at various temperatures, thawed overnight at 2 °C, and then placed into a germination chamber at 23 °C in the dark.

For each cultivar, 30 seeds were used. The initial weight of the 30 seeds was measured, and subsequently imbibed in deionized water for 24 hours at +2 °C, representing the typical soil temperature during fall-dormant seeding, ensuring conditions that mimic natural germination environments. After 24 hours, the final weight of the imbibed seeds was taken. The excess water on the surface of the seeds was removed using blotting paper. The seeds were then transferred to test tubes and placed in a refrigerated circulating bath (NESLAB Endocal, Albuquerque, New Mexico) with an initial temperature of -2 °C and maintained at this temperature for one hour. The seed surface was nucleated with shaved ice to initiate freezing. The temperature was then lowered at a rate of -2 °C per hour with sampling at 2 °C intervals until -12 °C to determine the lowest temperature that killed 50% of the imbibed seeds (LT_50_). Upon reaching the selected test temperatures, the samples were removed from the cold bath and thawed overnight at +2 °C. Thawed seeds were then germinated in Petri dishes lined with hydrated triple Whatman #1 filter paper, without free-standing water, at a constant temperature of +23 °C in darkness in the germination chamber (Powers Scientific, Inc., DS33SD, Ontario) (**Figure 1**). The freezing tests were replicated three times. The germination test was used to evaluate the LT_50_ of the seeds. The tetrazolium test was conducted to determine the viability of ungerminated seeds in the tests.

#### 2.3.2 Effect of prolonged freezing duration on survival of imbibed seeds (LD_50_)

As per the LT_50_ test, the initial weight of the 30 seeds was determined, and then imbibed in deionized water for 24 hours at +2 °C. After 24 hours, the final weight of the imbibed seeds was taken. The excess water on the surface of the seeds was removed using blotting paper. The seeds were then transferred to test tubes and placed in a refrigerated circulating bath (NESLAB Endocal, Albuquerque, New Mexico) with a set temperature of -2 °C based on the LT_50_ values. Seeds were nucleated with shaved ice and held at -2 °C for a total of 16 days with samples removed at four-day intervals and the duration of time at which 50% of the seeds were killed was evaluated (LD_50_).

After removing the seeds from the freezing bath, seeds were thawed overnight at +2 °C in the dark. The thawed seeds were placed onto hydrated triple-Whatman #1 filter paper in Petri dishes, without any standing water, and then placed in a germination chamber (Powers Scientific, Inc. DS33SD, Ontario) set at a constant temperature of 23 °C. The freezing treatments in each experiment were replicated three times. The germination test was used to determine the LD_50_ of the seeds, and a tetrazolium test was conducted to assess the viability of the ungerminated seeds.

#### 2.3.3 Tetrazolium test

The viability of ungerminated seeds for each line was assessed using the Tetrazolium (TZ) test, following the established protocols outlined in the AOSA/SCST Tetrazolium Testing Handbook (2010 Edition). After imbibing seeds in 1% TZ solution overnight, they were evaluated for viability based on the red staining pattern of the embryo and cotyledons.

#### 2.3.4 Experimental design and statistical analysis

In freezing experiments (LT_50_ and LD_50_ freezing tests), treatments were arranged as factorial combinations in a completely randomized design with three replicates. The factors in the short-term freeze test included six freezing temperatures and cultivars. The factors in the long-term freeze test included four freezing durations and three cultivars. Each replicate represented one test tube with 30 seeds. A linear mixed-effects model was used to evaluate the genotype and temperature on germination percentage. The LT_50_ and LD_50_ values were predicted using probit analysis in Minitab (Minitab Statistical software release 20, Minitab Inc., State College, PA, USA).

### 2.4 Seed trait analysis

#### 2.4.1 Mean seed weight

Mean seed weight was expressed as 1000-seed weight (TSW). The TSW was calculated using randomly selected 250 seeds for each replicate of each line/cultivar.

#### 2.4.2 Initial moisture content

The seed moisture was calculated using the gravimetric oven drying method for seed moisture content determination by drying the samples at 60 °C for 72 hours (Hay et al., 2023) in an oven (Thelco Model 16, Precision Scientific, USA). The seed moisture content was expressed relative to the dry weight in percentage (Equation 2). Moisture content was calculated as follows:

(2) $$MC\left(\%\right)=\frac{W_{I}-W_{D}}{W_{D}}*100$$

Where:

W = water absorption rate (%)
W_I_ = the initial weight of seeds
W_D_ = dry weight

#### 2.4.3 Seed coat to cotyledon ratio

Forty seeds from each line/cultivar were carefully dissected using forceps and blades to separate seed coats and cotyledons. The samples were then dried in the oven for 72 hours at 60 °C. After 72 hours, the pooled weights of the seed coat and cotyledon were measured using an analytical balance (Mettler, Toledo, Ohio).

#### 2.4.4 Surface area and volume

The surface area and volume for lentil seeds were calculated by using the relationships (Equations 3, 4) reported by Tang and Sokhansanj (1993) (Tang and Sokhansanj, 1993) as follows:

(3) $$S=2\pi \;\left(a^{2}+h^{2}\right)$$

(4) $$V=\frac{\pi h}{3}\;\left(3a^{2}+h^{2}\right)$$

Where:

S = the surface area of the lentil seeds,
V = the volume of the lentil seeds,
a = the average of the major and minor diameters of lentil seeds,
h = the half seed thickness.

The diameter and the half-thickness of seeds for all 38 lentil cultivars/lines were measured using the Leica dissecting Microscope at 1X magnification.

#### 2.4.5 Seed coat thickness

The seeds of all 38 lentil lines/cultivars were cut longitudinally from the end opposite the hilum through the length of the seed (Vieira et al., 2013) and 10 measurements of seed coat thickness per seed was assessed under the compound microscope (LEICA DFC 7000T) at 20X magnification.

### 2.5 Seed composition analysis

#### 2.5.1 Phenolic profiling of the seed coat using LC-HRMS

##### 2.5.1.1 Plant materials

Seeds from 14 lentil genotypes, selected based on water uptake amount and cotyledon color, were used for phenolic profiling of the seed coat (**Table 1**). The seeds were dehulled using an abrasive grain testing mill (Model TM05, Satake Engineering Co., Hiroshima, Japan) and the seed coats were separated from the dehulled product stream using a column blower (Seedburo Equipment Co., Des Plaines, IL, USA). The seed coats were stored at −80 °C until use.

**Table 1:** Detailed description of lentil genotypes used in this study.

Cotyledon colour and water uptake amount	Red cotyledon		Yellow cotyledon		Green cotyledon
High*	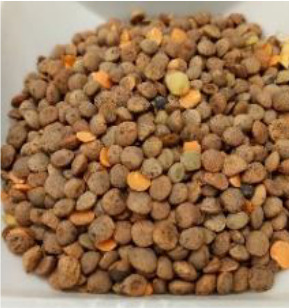 PI 251248 LSP (63.9%)	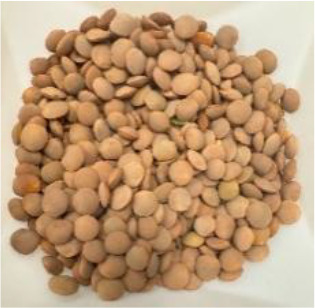 CDC KR-1 (73.1%)	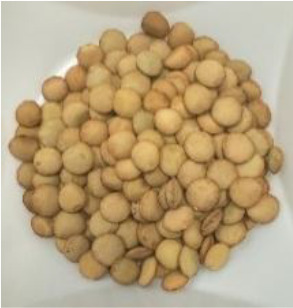 PI 298644 (69.6%)	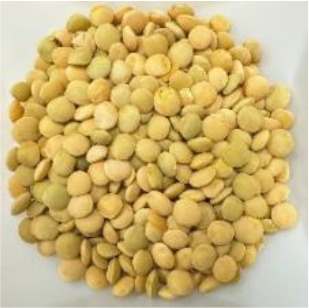 Shasta (79.3%)	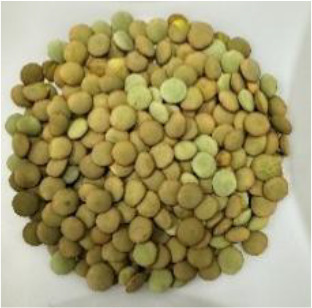 CDC QG-1 (68.4%)
Medium*	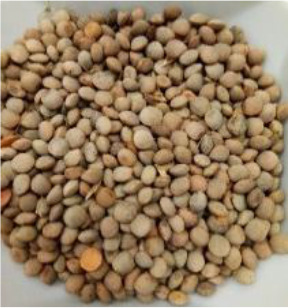 PI 193546 (63.6%)	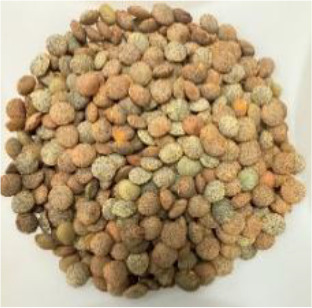 ILL 9997 (64.3%)	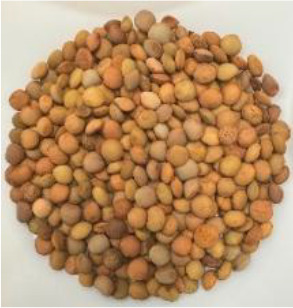 ILL 11548 (59.1%)	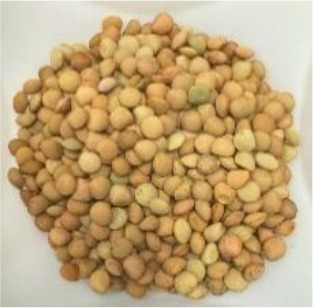 Eston (63.0%)	
Low*	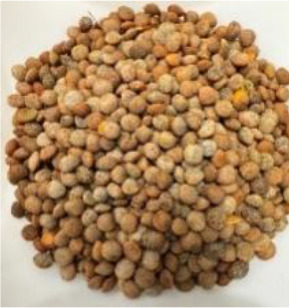 ILL 1983 (56.9%)	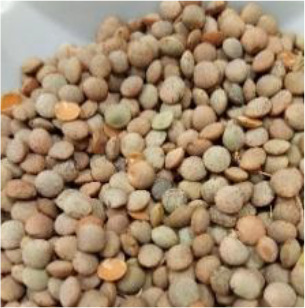 PI 320945 LSP (62.1%)	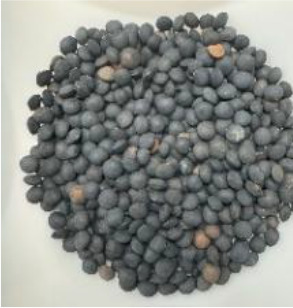 Indianhead (55.7%)	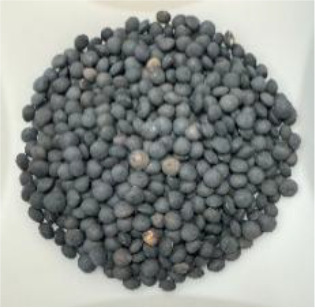 PI 320952 (58.0%)	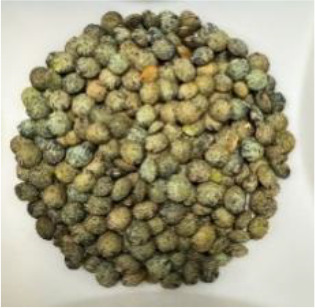 CDC Royale (67.7%)

* Low, medium, and high refer to categories of the lentil’s water uptake pattern. Numbers in brackets represent the final water absorption amount (%) after 24 hours at +2 °C.

##### 2.5.1.2 Preparation of seed coat extracts

Samples were prepared for untargeted analysis according to the extraction procedure of Elessawy et al. (2023) (Elessawy et al., 2023) with some modifications. In brief, 50 mg of each sample was placed into separate microcentrifuge tubes, stored in a −80 °C freezer for 1 hour, and then freeze-dried overnight at −80 °C at less than 0.133 mbar using a FreeZone Plus 6 freeze dryer (LabConco, Kansas City, MO, USA). The seed coats were pulverized to a fine powder using a mortar and pestle. A 1 mL aliquot of the extraction solvent, i.e., acetone (Thermo Fisher Scientific, Nepean, ON, Canada)/water (70:30 v/v) was added to the pulverized seed coats. After vortexing vigorously for 5-10 s, the samples were shaken for 1 hour at 23 °C on a Thermomixer C (Eppendorf, Hamburg, Germany) at 1,400 rpm. The samples were centrifuged at 16,200 × g for 10 min, and the supernatant was transferred into new, labelled tubes. The supernatants were centrifuged again at 16,200 × g for 5 min to ensure all the seed coat pellets were removed. A 200 μL aliquot of each extract was transferred to a new Eppendorf tube, dried down in a CentriVap vacuum concentrator (LabConco), and then reconstituted in 200 μL of MilliQ water/methanol (Thermo Fisher Scientific, Nepean, ON, Canada) (90:10 v/v). The reconstituted extract was transferred to a glass vial for analysis. Extraction efficiencies were not independently validated.

##### 2.5.1.3 Untargeted data acquisition of the extracts by LC-HRMS

The LC-HRMS instrumentation consisted of a Dionex 3000 LC coupled to a Quadrupole-Orbitrap (Thermo Fisher Q-Exactive) mass spectrometer, and a HESI (heated ESI) source was used. LC separation was achieved using a Waters HSS T3 column (2.1 × 100 mm, 1.8 μm) with a flow rate of 0.35 mL/min. A 30-minute run time was used, and the mobile phases were as follows: water/formic acid (99.9:0.1, v/v) as solvent A and water/acetonitrile (Fisher Scientific, Nepean, ON, Canada)/formic acid (9.9:90:0.1, v/v/v) as solvent B. After a 1 min hold at 1% B, gradient elution was performed according to the following conditions: from 1% B to 41% B in 20 min; 41 to 60% B in 4 min, 60 to 80% B in 0.1 min, hold at 80% B for 1.9 min, 80 to 1% B in 0.1 min, then hold at 1% B for 3.9 min. The quadrupole-Orbitrap (Thermo Fisher Q-Exactive) was used to acquire full scan data for the seed coat samples using a mass resolution (full width at half maximum, FWHM, @*m*/*z* 200) of 140,000 in negative mode with a mass range of 140–1800 *m*/*z*.

A quality control (QC) sample was prepared by taking an equal volume (20 µL) of supernatant from each replicate of the 56 seed coat samples (14 seed coat genotypes x 4 biological replicates) and mixing together in an Eppendorf tube labelled QC. The well mixed QC was added to a 2 mL amber vial and injected every 11 samples to account for changes in retention time and/or signal intensity, thereby allowing for relative quantification. The QC sample was also used to acquire data-dependent fragmentation data on the ions detected in full-scan mode using the scan function “Full scan/DDMS2”. Mass resolution of the full scan in the Full scan/DDMS2 analysis was 70,000 (FWHM @ m/z 200) and MS/MS was carried out on the 7 most abundant peaks at a resolution of 17,500 (FWHM @ m/z 200) using a stepped collision energy fragmentation. Three separate injections of the QC using Full scan/DDMS2 were done using collision energies of 10/20, 30/40, and 50/60 eV.

##### 2.5.1.4 Untargeted data analysis

A customized untargeted workflow, which has been described previously (Elessawy et al., 2023), was developed by adapting an existing workflow in the Thermo Fisher Compound Discoverer (CD) 3.3 software to process the LC-HRMS raw data. In brief, the Compound Discoverer workflow utilized full-scan accurate mass data to determine the possible molecular formula for each m/z value and used MS/MS spectra from ID samples to aid in compound identification. In addition to using Thermo’s mzCloud library, which contains fragmentation data of over 32,000 compounds analyzed with Thermo Orbitrap instrumentation (www.mzcloud.org), the MS/MS spectra were also compared (using the mzVault node) with those in an in-house library at the Core Mass Spectrometry Facility (University of Saskatchewan, Canada). Fragmentation spectra from several other libraries were also used offline, including libraries available in public databases, such as FoodB (foodB.ca), polyphenol-explorer (phenol-explorer.eu), and the human metabolome database (hmdb.ca). The identification levels followed those reported by Sumner et al. (2007) (Sumner et al., 2007), which include confirmed (1), putative (2), class only (3) and unidentified (4), with the addition of level (2/3) to indicate isomeric compounds as was reported in previous work (Elessawy et al., 2021). To focus on polyphenol detection, the results were filtered using a retention time window between 2 and 20 min (Elessawy et al., 2021).

Volcano plots, a type of differential analysis, were used to compare metabolites between two groups. The relative peak areas (calculated for each replicate within a group and the median value was used) needed to be ≥4.0 times different (log_2_ fold change = 2) and the *P*-value< 0.001 (>99.9% confidence) to be considered significant. *P*-values per group ratio were calculated by ANOVA and Tukey HSD *post hoc* tests.

#### 2.5.2 Determination of starch, amylose and protein content in the seed

##### 2.5.2.1 Plant materials

Seeds of six lentil lines/cultivars, [CDC KR-1 (large-seeded red cotyledon cultivar), PI 320945 LSP (extra small-seeded red cotyledon line), Shasta (large-seeded yellow cotyledon cultivar), Indianhead (extra small-seeded yellow cotyledon cultivar), CDC QG-1 (large-seeded green cotyledon cultivar) and CDC Royale (large-seeded green cotyledon cultivar)], were selected based on final water absorption rate.

##### 2.5.2.2 Sample preparation

Seeds of individual samples were de-hulled using a Satake TM05 lab-scale grain testing mill (Satake Corporation, Japan) equipped with a 40-mesh, grade P stone, rotating at 1,480 rpm. The lentil seeds were milled using Laboratory Mill 3100 (Perten Instruments Canada, Winnipeg, MB, Canada) to pass through an equipped 0.5-mm sieve to prepare lentil flour. All parts of the milling machine were cleaned after each sample to prevent cross-contamination between samples.

##### 2.5.2.3 Total starch and amylose content

Total starch content of the milled lentil flour samples was measured using a Megazyme Total Starch Assay Kit following AACC Method 76–13.01 (AACC, 2000). Amylose content was determined using an iodine colourimetric method (Li et al., 2021).

##### 2.5.2.4 Protein content

Protein content of the milled lentil flour samples was quantified as described by Liu et al. (2020) (Liu et al., 2020). In short, the Dumas combustion method was employed to measure their total nitrogen content using a Nitrogen/Protein Analyzer (CN628, LECO Corp., St. Joseph, MI, U.S.A.). Protein content was calculated by applying a nitrogen-to-protein conversion factor of 6.25 in accordance with AACC Method 46-30.01 (AACC, 2000).

### 2.6 Statistical analyses

Statistical significance was assessed using nested ANOVA, followed by multiple comparison tests with Tukey’s adjustment at a significance level of p< 0.05. Pearson correlation analysis assessed potential relationships between the final water absorption rate and the chemical components. The statistical analyses were performed using RStudio (Version 2024.04.1). As the primary objective of this study was to identify seed characteristics associated with water uptake, detailed ANOVA and Tukey test results are not presented in this study.

In addition, multivariate regression analysis and principal component analysis (PCA) were conducted to investigate the relationship between final water absorption and seed morphology in lentil seeds using RStudio (version 2023.06.2).

## 3 Results

### 3.1 Final quantity of water absorption

#### 3.1.1 At room temperature (23 ± °C)

**Figure 2:**
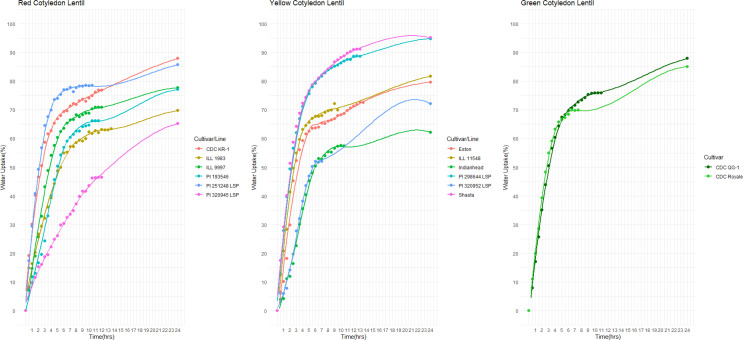
Water uptake (%) over time in red, yellow, and green cotyledon lentils at +23°C.

The final quantity of water absorption varied between 62.2% and 95.2% and the majority of water was absorbed within the first six hours (**Figure 2**). Notably, both the highest and lowest water absorption amounts were observed in yellow cotyledon lines. Shasta had the highest amount at 95.2%, while Indianhead had the lowest amount at 62.2% (**Figure 2**). Likewise, among cultivars/lines with red cotyledons, CDC KR-1 had the highest water absorption amount at 87.8%, while PI 320945 LSP had the lowest amount at 65.3%, suggesting that all lines/cultivars differed significantly in the final quantity of water absorption (p<0.05).

#### 3.1.2 Water uptake at 2 °C

**Figure 3:**
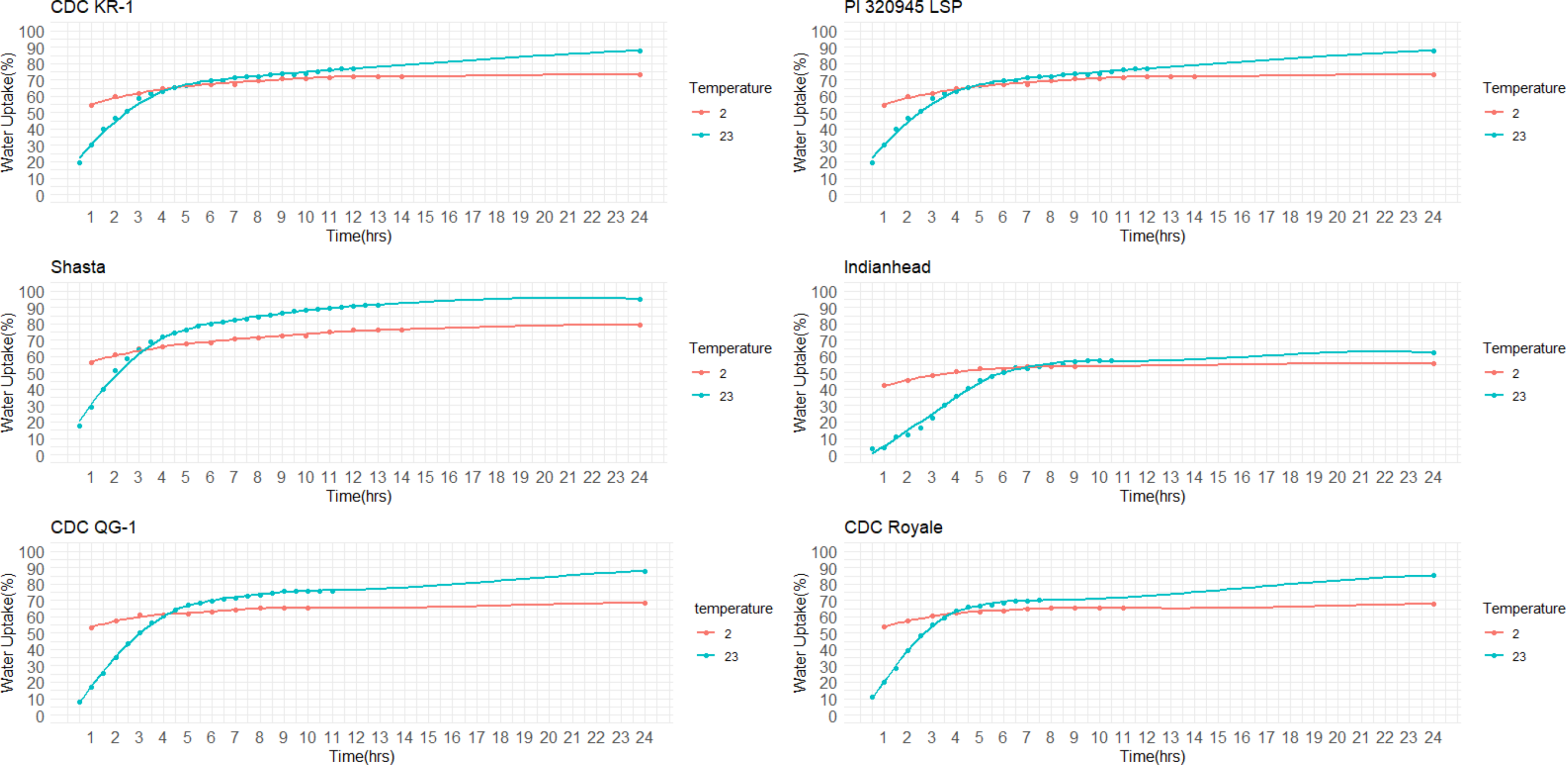
Effect of temperature on water uptake pattern in red (CDC KR-1, PI 320945 LSP), yellow (Shasta, Indianhead), and green (CDC QG-1, CDC Royale) cotyledon lentil lines/cultivars during a 24-hour period.

Among the 14 lentil lines/cultivars evaluated, all had rapid water absorption, increasing their weight by 43% to 55% within the first hour of imbibition (**Figure 3**). Despite differences in total water uptake, all lines/cultivars reached constant weight within a similar timeframe. By 24 hours, final water uptake varied significantly, ranging from approximately 55% to 80%, depending on cotyledon color and cultivar. Notably, both the highest and lowest water absorption rates were observed in yellow cotyledon lines. Shasta had the highest rate at 80%, whereas Indianhead had the lowest rate at 55%. Likewise, among cultivars/lines with red cotyledons, CDC KR-1 had the highest water absorption amount with 73%, while PI 320945 LSP had the lowest amount with 62.13%, suggesting that all lines/cultivars differed significantly in the final water absorption amounts.

### 3.2 Freezing tolerance of imbibed lentil seeds

#### 3.2.1 Freezing tolerance of imbibed seeds according to the LT_50_ test

Increasing temperatures during the freezing stress had a significant positive linear relationship with germination percentage (p< 0.05 and R^2^ 84%-98%). Imbibed seeds of all three lentil cultivars had an adverse impact on germination when the ambient temperature reached below -4 °C, at which temperature the seeds were killed. The average freezing tolerance (LT_50_) temperature for lentil was -3.2 °C (**Table 2**).

**Table 2:** LT_50_ for different cultivars/lines of lentil.

Crop species	Cotyledon colour	Cultivar/line	LT_50_(°C)
Lentil	Red	CDC KR-1	-4.8
	Yellow	Shasta	-2.6
	Green	CDC QG-1	-2.2

Some (5%) of the ungerminated seeds were contaminated with fungi or completely decayed, making them unsuitable for conducting the tetrazolium (TZ) test. However, non-contaminated but ungerminated seeds used for the TZ test confirmed that those seeds were not viable.

#### 3.2.2 The effect of time and temperature (LD_50_) on the freezing tolerance of seeds

Cultivar (C), duration that seeds were exposed to sub-zero temperature (D), and the C x D interaction had a significant effect on the germination percentage of lentil. LD_50_ values were cultivar specific: for example, 34 days for CDC KR-1, 15 days for CDC QG-1 and 0 days for Shasta (**Table 3**).

**Table 3:** Lethal duration at which 50% of the seeds were killed (LD_50_) for different cultivars of lentils.

Crop species	Cotyledon colour	Cultivar/line	LD_50_(days)	Temperatures of seeds were held (°C)
Lentil	Red	CDC KR-1	34	-2
	Yellow	Shasta	0	
	Green	CDC QG-1	15	

**Figure 4:**
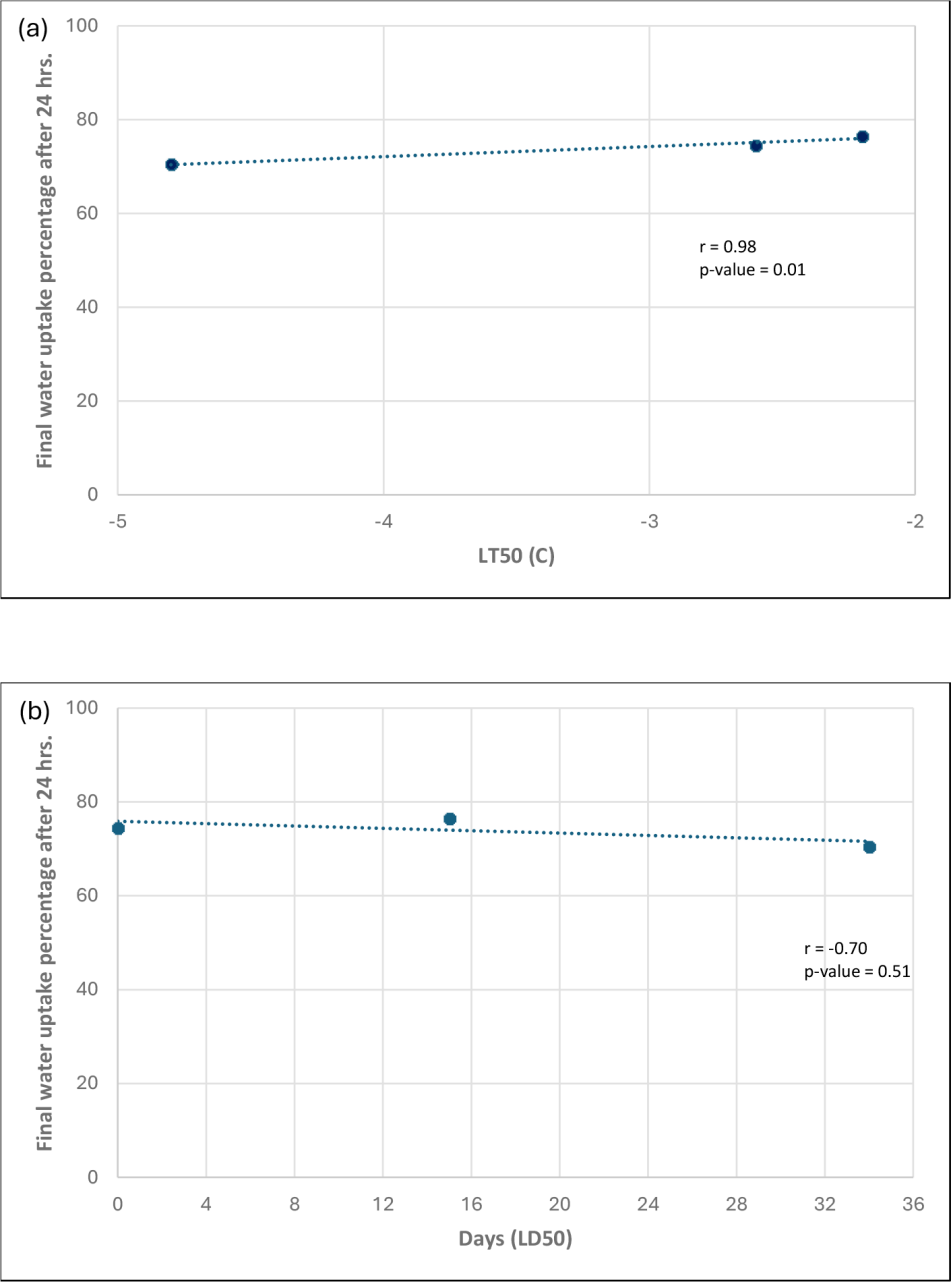
Correlation coefficient values for final water uptake percentage after 24 hours on three cultivars and **(a)** LT_50_, **(b)** LD_50_. Note: Correlation analysis is based on n=3 cultivars and three replicates. Additional genotypes are needed for robust statistical inference.

A strong positive correlation (r = 0.7) was observed between the final water absorption amount and LT_50_ freezing tolerance, suggesting that seeds with higher water absorption have lower freezing tolerance and vice versa (**Figure 4a**). In contrast, no significant relationship was found between final water absorption and LD_50_ (r = -0.3) (**Figure 4b**).

### 3.3 Variation in seed morphological and compositional traits among lentil lines/cultivars

Descriptive statistics for 12 seed traits were quantified across 38 lentil lines/cultivars (**Table 4**). Thousand seed weight (TSW) exhibited substantial phenotypic variation, ranging from 27.2 to 107.1 g (mean = 55.45 g; CV = 38.2%). Cotyledon weight (CW) and seed coat weight (SCW) also showed considerable variability, with CVs of 35.5% and 32.8%, respectively. In contrast, the seed coat-to-cotyledon ratio (SCCR) displayed relatively low variation (CV = 13.3%).

**Table 4:** Descriptive statistics of seed characteristics of 38 lentil lines/cultivars.

Seed characteristics	Maximum value	Minimum value	Mean	Standard deviation	Coefficient of variation (CV) (%)
AC	18.7	14.8	16.2	1.1	6.8
CW	74.4	20.8	38.8	13.8	35.5
FWAR	98.1	59.7	80	8.8	11
M	88.9	12.5	33.2	22.4	67.5
PC	33	25.7	29.2	2.3	7.9
SA	618.7	60.9	266.4	130.3	48.9
SC	48.1	42	44.9	2	4.5
SCCR	13.8	6.5	7.9	1.1	13.3
SCW	5.8	1.6	3	1	32.7
T	168.6	34.3	67.4	26.5	39.3
TSW	107.1	27.2	55.5	21.2	38.2
V	631.7	43.3	248.2	140.3	56.5

Amylose Content (AC), Cotyledon weight (CW), Final water absorption amount (FWAR), Initial moisture content (M), Protein Content (PC), Seed coat thickness (T), Seed coat to cotyledon ratio (SCCR), Seed coat weight (SCW), Starch Content (SC), Surface area (SA), Thousand Seed Weight (TSW), Volume (V).

Initial moisture content (M) demonstrated the greatest variability among all measured traits (CV = 67.5%). Seed coat thickness (T) also varied markedly (CV = 39.3%). Surface area (SA) and volume (V) were highly variable, with CVs of 48.9% and 56.5%, respectively. Final water absorption (FWAR) was comparatively stable across cultivars (CV = 11.0%).

Compositional traits showed limited variation: starch content (SC), amylose content (AC), and protein content (PC) had CVs of 4.5%, 6.8%, and 7.9%, respectively. Overall, morphological and physical seed traits exhibited extensive phenotypic diversity, whereas compositional traits remained relatively consistent across lentil lines, suggesting greater underlying genetic heterogeneity for structural characteristics than for biochemical composition.

### 3.4 Correlation analysis between seed characteristics and final water absorption amount in lentil seeds

**Figure 5:**
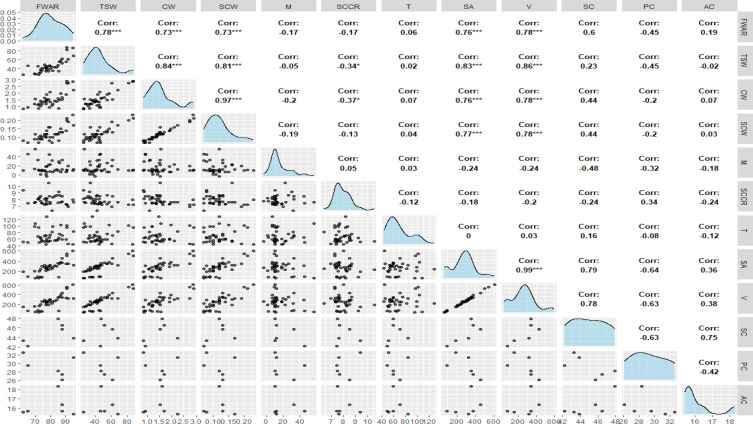
Correlation matrix with Pearson correlation coefficients (r) between different measured seed characteristics^1,2^. ^1^AC, Amylose Content; CW, Cotyledon weight; FWAR, Final water absorption amount; M, Initial moisture content; PC, Protein Content; T, Seed coat thickness; SCCR, Seed coat to cotyledon ratio; SCW, Seed coat weight; SC, Starch Content; SA, Surface area; TSW, Thousand Seed Weight; V, Volume. ^2^ *, and *** denote significant at p < 0.05 and 0.001, respectively; absence of a symbol indicates non-significant at p<0.05.

To investigate the association between FWAR and seed morphology in lentil seeds, a correlation analysis was performed using FWAR and seed characteristics (**Figure 5**).

FWAR had strong positive correlations with several morphological characteristics of seeds, including TSW (r = 0.78), CW (r = 0.73), SCW (r = 0.73), SA (r = 0.76), and V (r = 0.78). These findings suggest that larger and heavier seeds tend to absorb more water. Conversely, FWAR was weakly negatively correlated with M (r = -0.17) and SCCR (r = -0.17), indicating that seeds with higher initial water content or proportionally thicker seed coats relative to cotyledons may have reduced water absorption efficiency. PC also showed a moderate negative correlation (r = -0.45). In contrast, SC had a moderate positive correlation (r = 0.60). T (r = 0.06) and AC (r = 0.19) showed no strong correlations with FWAR.

### 3.5 Relationship between seed characteristics and the final water absorption amount

A multiple regression analysis was conducted to evaluate the relationship between lentil seed characteristics and FWAR. Seven variables were selected based on multicollinearity diagnostics from an initial set of 11 independent variables, specifically ensuring that each retained variable’s Variance Inflation Factor (VIF) was less than 7. This helped to ensure a robust and interpretable model by reducing redundancy and collinearity among predictors.

Utilizing the multi-variate regression method, the relationship Equation 5 linking the final water absorption rate to seed characteristics is formulated as follows:

(5) $$\text{FWAR}=38.06+0.3{\text{X}}_{TSW}-0.03{\text{X}}_{M}+0.02{\text{X}}_{T}+0.01{\text{X}}_{SA}+0.93{\text{X}}_{SC}-0.18{\text{X}}_{AC}-0.78{\text{X}}_{PC}$$

(R^2^-value = 0.84**; p-value = 2.164e-06**)

The regression model revealed a high coefficient of determination (R^2^ = 0.84), indicating the selected seed characteristics explained 84% of the variation in the FWAR. The model was statistically significant (p = 2.164 × 10^-6^), reflecting a strong overall fit.

Among the variables, SC had the most significant positive effect on FWAR (β = 0.93), suggesting that seeds with higher total starch levels tend to absorb more water. TSW also had a substantial positive relationship (β = 0.30), indicating that larger seeds absorb water more readily, possibly due to increased internal space and surface contact. Minor positive effects were observed for T (β = 0.02) and SA (β = 0.01), implying that structural properties contribute to the water uptake process, albeit to a lesser extent.

Conversely, PC had the most negative impact on FWAR (β = -0.78), indicating that higher protein levels reduce water absorption capacity. M (β = -0.03) also negatively influenced FWAR suggesting that seeds with pre-existing moisture absorb less additional water during hydration.

These findings suggest that structural and chemical properties significantly affect the final water absorption of lentil seeds, with starch and protein content being the most influencing factors.

### 3.6 Principal component analysis of seed characteristics and final water absorption amount of lentil seeds

**Figure 6:**
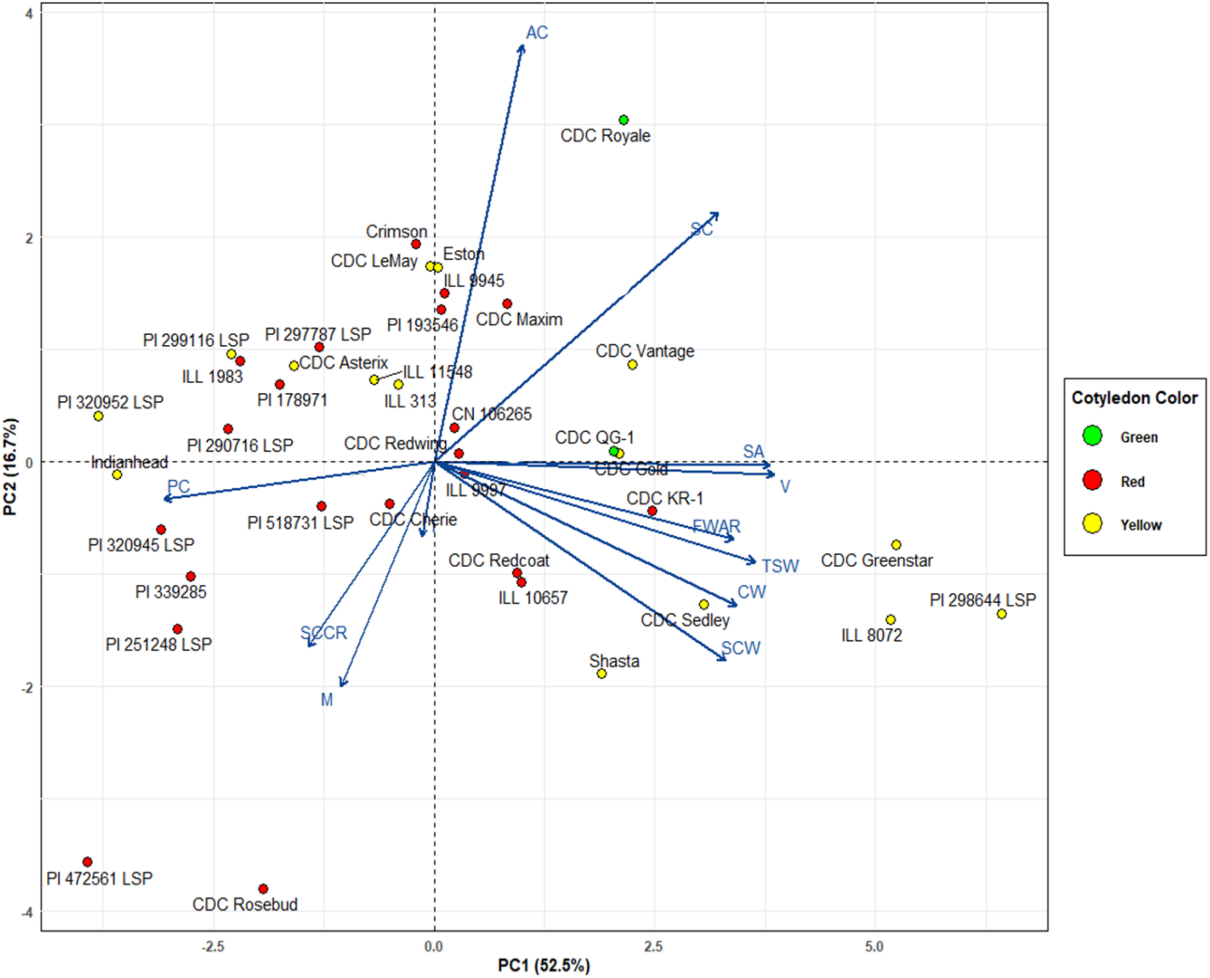
Biplot between PC1 and PC2 showing contribution of twelve traits in variability of 38 genotypes.

The Principal Component Analysis (PCA) is performed to help understand the underlying data structure and/or form a smaller number of uncorrelated variables, which would avoid multicollinearity in regression. We used PCA for this purpose using the 38 lentil genotypes based on 12 seed morphological and compositional traits. The first two principal components, PC1 and PC2, explained 52.5% and 16.7% of the total variation, respectively, accounting for a cumulative 69.2% of the total variability. A biplot of genotypes and trait loadings was plotted to facilitate interpreting trait relationships and genotype distribution (**Figure 6**).

Trait vectors indicated PC1 was strongly and positively influenced by TSW, CW, SA, V, and FWAR. PC2 was influenced by AC and PC, reflecting compositional variation.

Genotypes distributed along the positive axis of PC1 (e.g., CDC Greenstar, PI 298644 LSP, ILL 8072) were associated with larger seed size and higher FWAR. Those located on the negative side of PC1, particularly in the lower left quadrant (e.g. PI 472561 LSP, PI 320945 LSP, PI 339285), were characterized by reduced seed size, lower water absorption capacity and a potentially greater seed coat to cotyledon ratio, as indicated by their proximity to T and SCCR trait vectors. Genotypes in the upper left quadrant (e.g., CDC Asterix, CDC Rosebud) had intermediate profiles, distant from both the TSW/FWAR and composition vectors, suggesting moderate trait values.

Some degree of grouping based on cotyledon color was evident, although not strongly clustered. Several red cotyledon genotypes appeared in the left half of the biplot and were associated with reduced seed size and lower water absorption traits. Yellow cotyledon genotypes, such as CDC Greenstar and PI 298644 LSP were located toward the right, associated with greater seed volume and FWAR. Green cotyledon genotypes were more broadly distributed, with some positioned closer to the upper region of the plot, near vectors for protein and amylose content. These spatial patterns suggest that while cotyledon color may coincide with specific trait trends, it does not define discrete phenotypic clusters.

### 3.7 Phenolic profiling in lentil seed coats

**Figure 7:**
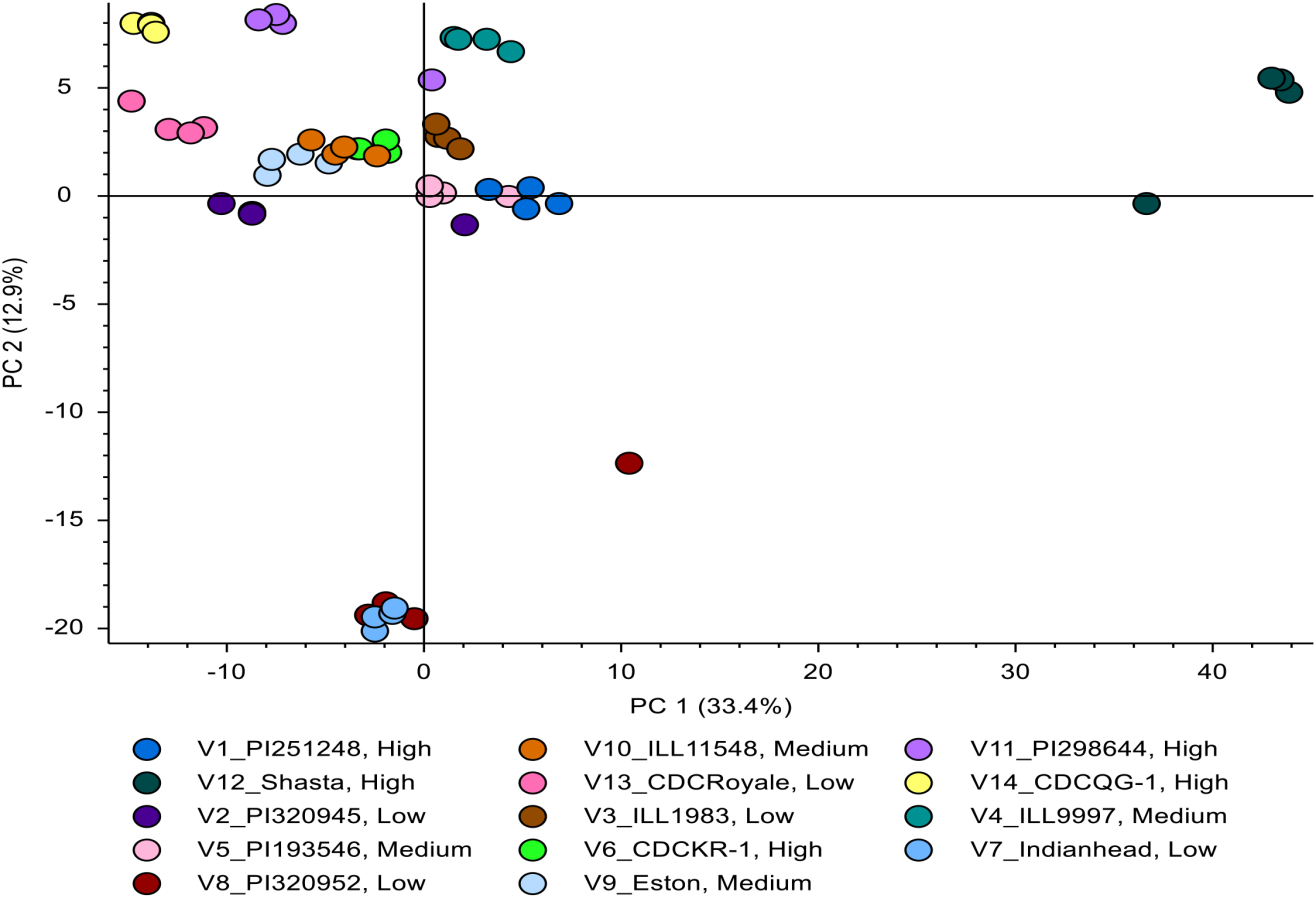
PCA plot using 516 compounds of low-, medium- and high-water uptake lentil seed groups*. *Low, medium, and high-water uptake groups were classified based on the final water absorption after 24 h at ±2 °C.

**Figure 8:**
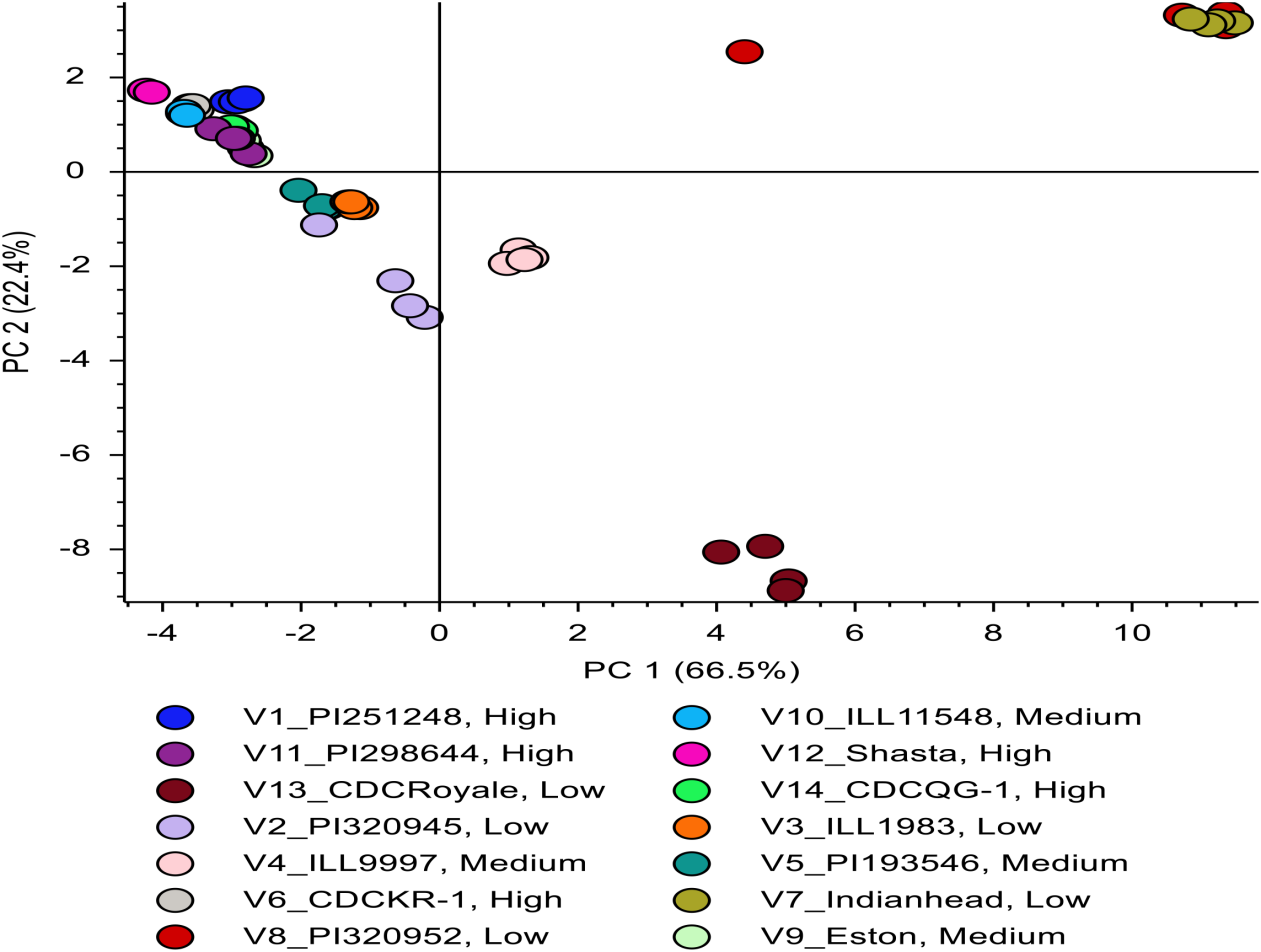
PCA plot using 36 compounds of low-, medium- and high-water uptake lentil seed groups*. *Low, medium, and high-water uptake groups were classified based on the final water absorption after 24 h at ±2 °C.

**Figure 9:**
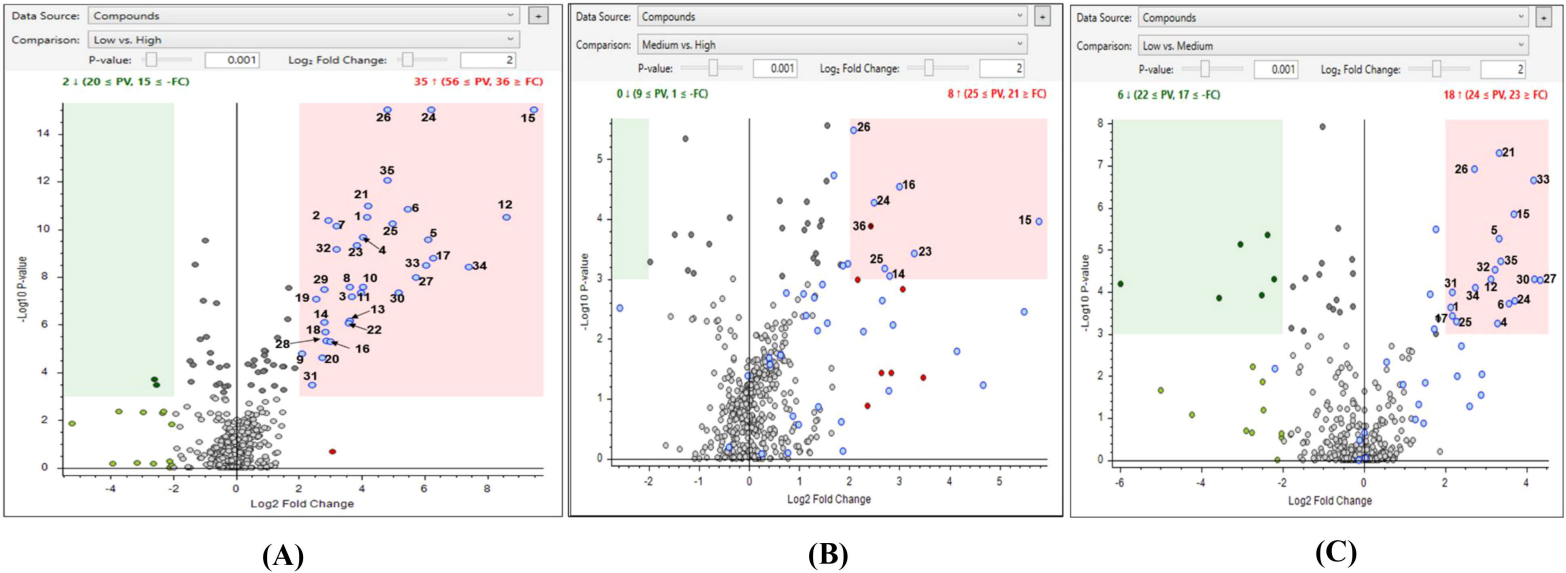
Volcano plots comparing lentil seed coats from low-, medium-, and high-water uptake groups are shown for low vs. high **(A)**, medium vs. high **(B)**, and low vs. medium **(C)** water uptake characteristics.

Phenolic profiling was conducted on a subset of 14 genotypes representing water uptake profiles; therefore, these results should be interpreted cautiously and may not fully represent phenolic variation across all 38 genotypes evaluated in this study. LC-HRMS untargeted metabolomics was used to explore the polyphenolic variations among lentil seed coats with different water uptake patterns. Initially, lentil seed coat genotypes with low water uptake were compared to those with high and medium water uptake. Compound Discoverer analysis of the raw data generated a list of metabolites, which was used to create a PCA plot of seed coats (**Figure 7**), whereas **Figure 8** focuses only on the 36 upregulated compounds identified in the volcano plots (**Figure 9**).

High- and medium-water uptake genotypes clustered closely in the PCA, indicating relatively minor differences in their polyphenolic compositions (**Figure 7**). Low-uptake genotypes were more dispersed, with the low-tannin genotype ‘Shasta’ forming a distinct outlier relative to all tannin-containing lines. Because tannins comprise a major fraction of lentil seed coats, Shasta’s low-tannin, thinner seed coat likely contributes to its exceptionally high final water absorption. This is supported by a strong positive correlation between Shasta’s seed coat thickness and water uptake (r = 0.98).

Dark-seeded genotypes (PI 320952 and Indianhead) also exhibited polyphenolic profiles distinct from lighter-colored genotypes, consistent with their relatively low water uptake (58% and 56%). The contrasting positions of Shasta and the dark-colored genotypes in the PCA plot suggest that variation in polyphenol composition—particularly tannins—plays an important role in hydration behavior.

**Figure 10:**
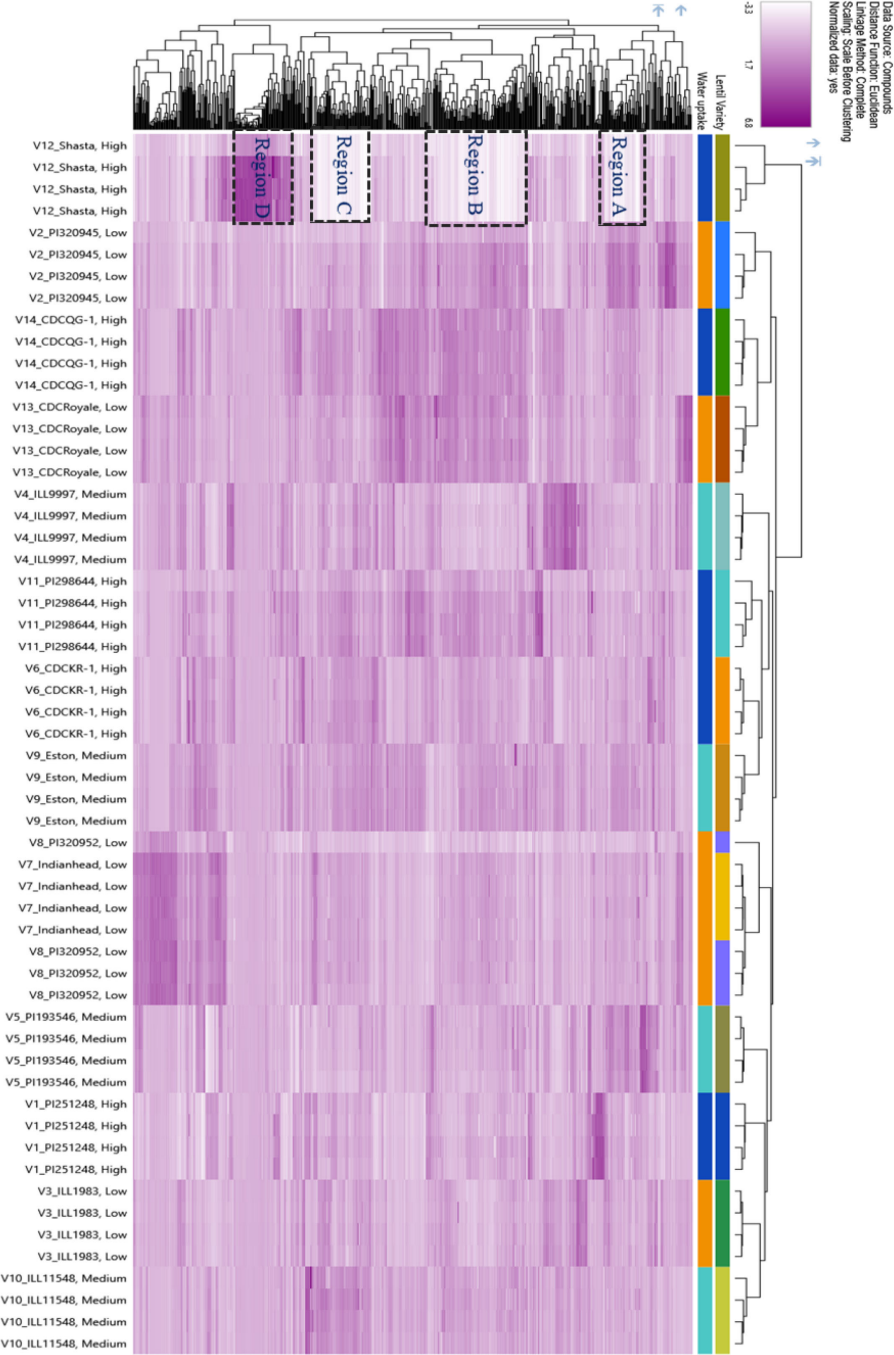
HCA plot of lentil seed coat samples grouped into high, medium, and low water uptake categories based on 516 metabolites identified in the volcano plots. Each rectangle* represents a metabolite, with color intensity corresponding to the relative abundance (by area) of that metabolite in each sample. *Region A comprises 33 compounds, predominantly proanthocyanidins (31 compounds), along with one amino acid derivative and one phenolic acid derivative. Region B contains 82 compounds, mainly proanthocyanidins (77 compounds), in addition to two flavonols, two hydroxybenzoic acid derivatives, and one unknown C-glycoside. Region C includes 48 compounds, all of which are proanthocyanidins. Region D consists of 53 compounds, characterized by flavonols, fatty acid derivatives, phenolic acids, terpenoid glycosides, chalcones, and several unknown compounds.

Hierarchical cluster analysis (HCA) (**Figure 10**) further confirmed differences in polyphenolic composition. Proanthocyanidins (regions A–C) were abundant in tannin-rich genotypes but nearly absent in Shasta, while compounds in region D—primarily kaempferol-based flavonols—were more abundant in Shasta. As an extreme low-tannin genotype, Shasta consistently showed the highest water absorption.

Seed-coat color also corresponded with phenolic profiles: black-seeded lines (‘Indianhead’, ‘PI 320952’) grouped separately from dark green or marbled genotypes such as ‘CDC Royale’. Genotypes with similar seed-coat colors or patterns clustered together in **Figure 7**, supporting the linkage between pigmentation, polyphenol composition, and water uptake.

Volcano plot analysis (**Figure 9**; p< 0.001, log_2_ fold change > 2) identified metabolites differing significantly across low-, medium-, and high-water uptake groups. Metabolites enriched in low-uptake genotypes appeared to the right of zero (**Figures 9A, C**), whereas those enriched in high- or medium-uptake groups appeared to the left. Most metabolites showed no significant abundance differences, but those in the highlighted regions met both significance thresholds. Blue-colored points in these regions represent metabolites elevated in low-uptake genotypes (or in medium relative to high), suggesting they may be key regulators of water uptake in lentils.

Using a p-value of 0.001 and a log_2_ fold change of 2 (indicating a four-fold change), the upregulated and downregulated metabolites are highlighted in pink and green areas, respectively. Additionally, blue dots represent metabolites that are significantly higher in the low water uptake group compared to the medium and high-water uptake groups. The metabolite numbers correspond to those listed in **Table 5**.

**Table 5:** Identification of the 35 upregulated compounds^λ^ (top 10 compounds by area in bold) shown in the volcano plot Low vs High (**Figure 9A**).

No.	Name	Formula	Calculate molecular weight (g/mol)	RT [min]	Mass error (ppm)	Identification level
1*	**Tricetin**	**C_15_H_10_O_7_**	**302.0426**	**16.21**	**-0.19**	**1**
**2**	**Luteolin**	**C_15_H_10_O_6_**	**286.0477**	**18.46**	**-0.26**	**1**
**3**	**Tricetin hexoside**	**C_21_H_20_O_12_**	**464.0958**	**14.13**	**0.68**	**2/3**
4*	**Luteolin hexoside**	**C_21_H_20_O_11_**	**448.1008**	**15.08**	**0.44**	**2/3**
5*	**Delphinidin 3-O-glucopyranosyl-arabinopyranoside**	**C_26_H_28_O_16_**	**596.138364**	**8.79**	**1.04**	**2**
6*	**Tricetin hexoside**	**C_21_H_20_O_12_**	**464.0958**	**15.92**	**0.75**	**2/3**
7	Tricetin derivative	C_33_H_34_O_23_	798.1508	14.96	2.13	3
**8**	**Luteolin hexoside**	**C_21_H_20_O_11_**	**448.1010**	**15.83**	**1.03**	**2/3**
**9**	**Quercetin 3,4’-di-O-glucoside**	**C_27_H_30_O_17_**	**626.1492**	**13.41**	**1.47**	**2**
10	Luteolin acetylhexoside	C_23_H_22_O_12_	490.1047	16.58	1.73	2/3
**11**	**Luteolin malonylhexoside**	**C_24_H_22_O_14_**	**534.1013**	**16.96**	**0.66**	**2/3**
12*	**Trihydroxycoumarin pentoside hexoside**	**C_20_H_24_O_14_**	**488.1171**	**7.25**	**1.01**	**3**
13	Tricetin malonylhexoside	C_24_H_22_O_15_	550.0967	15.17	1.46	2/3
14^ϯ^	Luteolin malonylhexoside	C_24_H_22_O_14_	534.1018	15.45	1.59	2/3
15*^ϯ^	Heliotropic acid gallate	C_15_ H_10_O_8_	318.03784	14.60	0.6	3
16^ϯ^	Unknown C-glycoside	C_26_H_32_O_14_	568.1799	15.19	1.27	4
17*	Tricetin pentoside	C_20_H_18_O_11_	434.0853	12.59	0.9	2/3
18	{5-[(D-Galactopyranosyloxy)methyl]-2-furyl}methyl D-galactopyranoside	C_18_H_28_O_13_	452.1536	2.07	1.23	3
19	Myricetin dihexoside	C_27_H_30_O_18_	642.1440	10.44	1.18	2/3
20	Luteolin malonylhexoside	C_24_H_22_O_14_	534.1017	16.80	1.4	2/3
21*	Myricetin pentoside hexoside	C_26_H_28_O_17_	612.1335	12.21	1.33	2/3
22	Isorhamnetin hexoside	C_22_H_22_O_12_	478.1118	15.78	1.4	2/3
23^ϯ^	Luteolin hexoside	C_21_H_20_O_11_	448.1009	13.59	0.65	2/3
24*^ϯ^	Tricetin malonylhexoside	C_24_H_22_O_15_	550.0967	17.39	1.44	2/3
25*^ϯ^	Tricetin hexoside	C_21_H_20_O_12_	464.0959	11.95	0.98	2/3
26*^ϯ^	Gossypetin	C_15_H_10_O_8_	318.0376	12.77	0.18	3
27*	Monoxerutin or isomer	C_29_H_34_O_17_	654.1806	10.41	1.57	2/3
28	Apigenin C-hexoside C-pentoside	C_26_H_28_O_14_	564.1485	11.76	0.98	2/3
29	Isorhamnetin hexoside	C_22_H_22_O_12_	478.1118	16.14	1.3	2/3
30*	Monoxerutin or isomer	C_29_H_34_O_17_	654.1806	9.74	1.55	2/3
31*	Trihydroxy-megastigmadiene-one-hexoside derivative	C_25_H_38_O_13_	546.2322	15.32	1.66	3
32*	Isorhamnetin isomer	C_16_H_12_O_7_	316.0588	18.66	1.51	2/3
33*	Delphinidin derivative	C_32_H_40_O_22_	776.2022	7.06	1.33	3
34*	Luteoin pentoside	C_20_H_18_O_10_	418.0907	14.39	1.78	2/3
35*	Unknown Flavonoid	C_15_H_8_O_8_	316.0223	9.44	1.11	3
36 ^λ^	Unknown fatty acid	C_30_H_50_O_18_	698.3008	17.73	1.54	3

Bold compounds have the top 10 max. peak areas among the samples.

^λ^Compound 36 only upregulated in medium vs. high volcano plot (not low vs. high).

*Compound also upregulated in Low vs. Medium volcano plot.

^ϯ^Compound also upregulated compounds in Medium vs. High volcano plot.

Identification levels are confirmed (1), putative (2), isomeric (2/3), class only (3) and unidentified (4). The identification levels in the table follow Sumner et al (Sumner et al., 2007)with the addition of level 2/3 indicating isomeric compounds (Elessawy et al., 2021).

## 4 Discussion

Seed water uptake amount varied among lentil cultivars, and temperature significantly affected both the rate and extent of water uptake. Seeds imbibed at 2 °C absorbed water more rapidly during the first hour which is consistent with the findings in soybean seeds in which the increased temperatures increased total water uptake (Hsu et al., 1983), further supporting the role of temperature in influencing both the rate and extent of seed hydration. The initial rapid imbibition at low temperature reflects a steep water potential gradient across the seed (Vertucci and Leopold, 1983), while the lower overall absorption exhibits reduced diffusion rates and metabolic activity in cold conditions (Hsu et al., 1983). Moisture content (MC) directly shapes freezing tolerance of a seed. Our findings agree with earlier work in lettuce and other crops showing that seeds with low MC (<13%) maintain high viability at ultra-low temperatures, whereas higher MC promotes intracellular ice formation and injury (Junttila and Stushnoff, 1977; Keefe and Moore, 1981; Roos and Stanwood, 1981). Excess hydration increases the likelihood of intracellular freezing, disrupting membranes and reducing survival (Jaganathan et al., 2020), consistent with a decline in freezing tolerance with increasing MC in our study.

Freezing injury differs between short-term (LT_50_) and prolonged (LD_50_) exposure. The plasma membrane is the primary site of freezing stress perception (Yamazaki et al., 2009), as cold temperatures reduce membrane fluidity and trigger the leakage of ions and metabolites (Levitt, 1980; Knight et al., 1998; Seo et al., 2010). Short exposures cause reversible injury, but prolonged freezing induces desiccation, protein denaturation, enzyme inactivation, and oxidative stress (Mahajan and Tuteja, 2005; Solanke and Sharma, 2008; Theocharis et al., 2012; Kumar, 2018; Georgieva et al., 2022). In our study, the lack of correlation between MC and LD_50_ suggests that long-term freezing damage in lentils is dominated by desiccation and solute concentration effects, rather than water content alone which aligns with models where “solution effects” stresses caused by ion concentration during freezing drive injury more strongly than total water content (Meryman et al., 1977; Nagao et al., 2007).

On average, seed morphological traits showed strong correlations with water uptake amount. Thousand-seed weight, surface area, and volume all increased water absorption, consistent with results in lentil (Al-Karaki, 1998), safflower (Farhoudi and Motamedi, 2010) and soybean (Burris et al., 1971). These traits likely enhance seed–water contact and reflect higher starch reserves that promote osmotic water movement (Kikuzawa and Koyama, 1999; Albert et al., 2024). Initial moisture content, by contrast, had little effect on imbibition (r = -0.18). Similar results were observed in tomato and bean (Badek et al., 2006), indicating that internal water potential contributes minimally to imbibition (McDonald et al., 1988). Contrary to patterns in soybean (Noodén et al., 1985; Jang et al., 2015), common bean (Wyatt, 1977; Balasubramanian et al., 2004), and species of *Medicago* and *Trifolium* (Russi et al., 1992), lentil seed coat thickness was unrelated to water absorption amount. Similar independence was reported in peas (*Pisum sativum*) (Hradilová et al., 2019; Williams et al., 2024) and lentils (Guerra-García et al., 2025). However, a strong positive relationship was observed between seed coat thickness and FWAR (r = 0.98), in Shasta, supporting the role of seed coat thickness in influencing water absorption within this genetic background. Low-tannin genotypes typically possess thinner seed coats, which may explain why Shasta exhibits the highest final water absorption amount. However, when all 38 genotypes were analyzed together, the correlation was weak and not significant, likely due to substantial genotypic variability in seed coat properties, such as tannin composition, microstructure, and permeability that influence FWAR independently of thickness. Therefore, one possible explanation for the lack of correlation between water uptake amount and seed coat thickness in lentils in general may lie in the biochemical composition of the seed coat, particularly its phenolic content. Our metabolomic analyses revealed distinct clustering of lines/cultivars based on water uptake and phenolic content, with low-uptake lines/cultivars separated from medium and high-uptake types in the PCA plots (**Figure 7**). This pattern aligns with previous findings that polyphenolic compounds are critical for seed coat impermeability (Marbach and Mayer, 1974; Werker et al., 1979; Mohamed-Yasseen et al., 1994). Particularly notable is the low-tannin cultivar “Shasta”, which had the highest water absorption and a lower abundance of proanthocyanidins (also called condensed tannins). This observation supports earlier reports that tannins and related polyphenols reduce seed coat permeability (Marbach and Mayer, 1974; Debeaujon et al., 2000). However, the relationship between phenolic composition and freezing tolerance remains to be directly tested.

Volcano plot analyses (**Figure 9**) revealed that several flavones, including tricetin, tricetin malonylhexoside, and delphinidin derivatives, were significantly upregulated in low water uptake lines. Among these, tricetin and its derivatives form water-insoluble phenolic–polysaccharide complexes that can inhibit water entry (Bentsink and Koornneef, 2008). The accumulation of such compounds in the seed coat likely contributes to the formation of a dense, phenolic-rich cuticle that serves as a barrier to water, consistent with previous studies suggesting that these secondary metabolites delay or prevent imbibition by limiting porosity or increasing surface hydrophobicity (Smýkal et al., 2014).

The HCA plot (**Figure 10**) further differentiated ‘Shasta’ and other lines based on the abundance of specific polyphenolic subclasses, including proanthocyanidins and flavonols, including kaempferol glycosides. These compounds are known to participate in oxidative polymerization, enhancing cell wall rigidity and hydrophobicity (Lepiniec et al., 2006; Pourcel et al., 2007). Supporting this, studies in *Arabidopsis* have shown that mutants lacking genes involved in flavonoid biosynthesis produce thinner and more permeable seed coats (Lepiniec et al., 2006). In legumes, water-impermeable seed dormancy has long been attributed to the presence of flavonoids in the seed coat (Wyatt, 1977; Werker et al., 1979; Kantar et al., 1996).

Protein content showed a moderate negative correlation (r = -0.45) with water uptake, indicating that lentil seeds with higher protein levels absorbed less water. This finding contrasts with previous studies on species such as *Acacia polyphylla* and *Guazuma ulmifolia*, where high protein content has been shown to enhance water absorption due to the hydrophilic nature of proteins (Benech-Arnold and Rodolfo, 2004; Brancalion et al., 2008), which might be due to structural differences in protein localization or interactions within the seed matrix that restrict water penetration. Supporting this interpretation, Agbo et al. (1987) reported that seeds with dense protein matrices had greater resistance to water movement in dry beans (Agbo et al., 1987). If similar structural traits exist in lentil seeds, proteins may play a more barrier-forming role than a water-attracting one, hindering rather than facilitating imbibition. In contrast, total starch content showed a moderate positive correlation (r = 0.6) with water uptake, suggesting that starch-rich lentil seeds absorbed more water. Although Zhao et al. (2018) found that high-starch-containing seeds, such as *Chloris virgata* (Zhao et al., 2018), had reduced water uptake, our findings in lentils demonstrate an opposing trend, suggesting that species-specific anatomical and biochemical characteristics likely modulate the influence of starch on imbibition. In lentils, starch may be more loosely packed or less structurally integrated with protein, allowing it to interact more effectively with water during imbibition. These interpretations require validation through direct measurement of protein localization and starch granule packing density.

## 5 Conclusions

In summary, these findings suggest a model in which seed morphology and biochemistry of lentils converge to regulate water uptake and freezing tolerance. Larger, starch-rich lentil seeds absorb more moisture and are more susceptible to freezing damage. Smaller, phenolic- and protein-rich lentil seeds absorb water slowly and can retain their structural integrity under freezing conditions. These dual pathways, morphological and biochemical, determine overwinter survival. Both structural and biochemical traits can be targeted to select or breed lentil varieties with improved overwinter survival. Such improvements could enable reliable fall seeding of lentils, helping to extend the growing season and mitigate early-spring production risks on the Canadian prairies.

## Data Availability

The original contributions presented in the study are included in the article/**Supplementary Material**, further inquiries can be directed to the corresponding author/s.
