# Supplementary material for: Assessing seed characteristics for improved winter survival of late-fall-seeded lentils
Source: Front Plant Sci. 2026 Apr 2;17:1802566. doi: 10.3389/fpls.2026.1802566 (PMC13083018; doi:10.3389/fpls.2026.1802566)
Supplement: Supplementary file 1 [file Supplementaryfile1.pdf]

Supplementary Materials

1 Supplementary Tables

Supplementary Table 1. Lentil lines/cultivars used in the study

Cotyledon Color \ Size	Extra Small	Small	Medium	Large
**Red**	1.**PI 472561 LSP** (LD)<br>2.**PI 193546** (LC)<br>3.**PI 297787 LSP** (LP, LD)<br>4.**ILL 1983** (LP, LD)	1.**ILL 945** (HP)<br>2.**PI 178971 LSP** (HC)<br>3.**PI 518731 LSP** (LH)<br>4.**Crimson** (L1000, MP, MD, MC, HP, HC)<br>5.**CDC Redcoat** (MP, MD, MC, MH)<br>6.**PI 320945 LSP** (LH)<br>7.**CDC KR-1** (MP, M1000, MH)<br>8.**CDC Maxim** (MD, M1000, MC, MH, HC)<br>9.**CDC Rosebud** (L1000, MP, MC)<br>10.**CDC Cherie** (MD, M1000, MH, HC)<br>11.**CDC Redwing** (L1000, MP, MD, MC)	1.**ILL 9997** (HP)<br>2.**PI 290716 LSP** (L1000, HC)<br>3.**PI 251248 LSP** (L1000)<br>4.**CN 106265** (HP, HC)	1.**PI 339285** (HH)<br>2.**ILL 10657** (HP)
**Yellow**	1.**PI 320952 LSP** (L1000, LC)<br>2.**PI 299116 LSP** (LC)<br>3.**Indianhead** (LP, LD, LC, LH)<br>4.**ILL 313** (LD)	1.**CDC GOLD** (MD, M1000, HP, HH)<br>2.**CDC Asterix** (MD, M1000, HP, HH)<br>3.**CDC LeMay** (LP, LH, MD)<br>4.**Eston** (MP, MD, M1000, MC)	1.**ILL 11548** (L1000, HC)<br>2.**PI 298644 LSP** (LC)<br>3.**CDC Vantage** (MD, M1000, MH, HP, HC)<br>4.**CDC Sedley** (MP, M1000, MH, HP, HC)<br>5. **CN 106265** (HP, HC)	1.**ILL 8072** (H1000)<br>2.**CDC Greenstar** (MP, MC, MH, H1000, HH)<br>3. **Shasta** (MP, MC, HH)
**Green**				1.**CDC QG-1** (LH, HP)<br>2.**CDC Royale** (LH, MD, M1000, HP)

Note: L-Low, M-Medium, H-High
C-Circularity, D-Diameter, H-Height, P-Plumpness, and 1000- thousand-seed weight.

Supplementary Table 2. Representative Images of Seeds from Different Genotypes

Red cotyledon lentil				
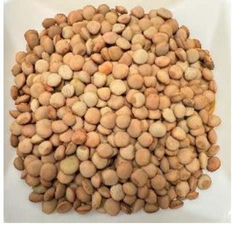 Crimson	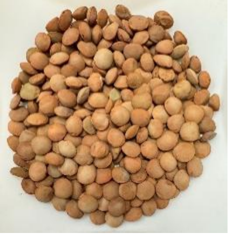 ILL 10657	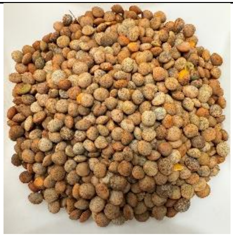 ILL 1983	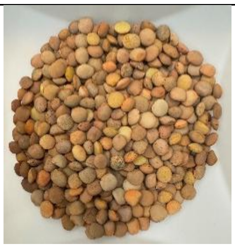 PI 290716 LSP	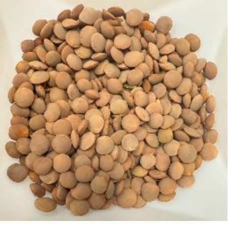 CDC KR-1
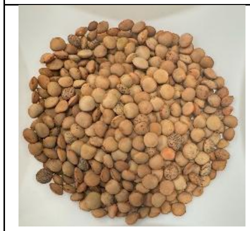 PI 518731 LSP	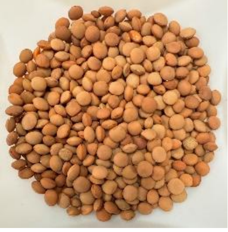 ILL 9945	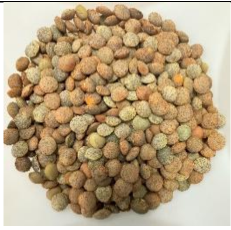 ILL 9997	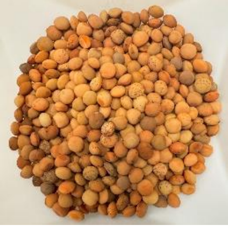 PI 339285	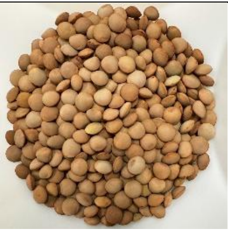 CDC Redcoat
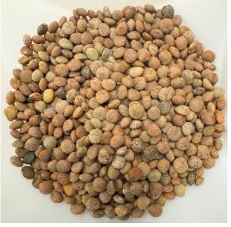 PI 472561 LSP	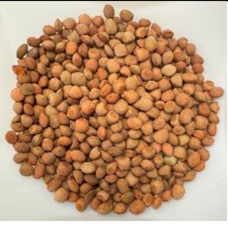 PI 297787 LSP	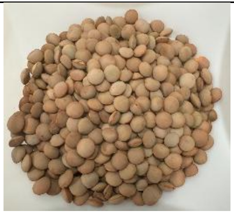 CDC Cherie	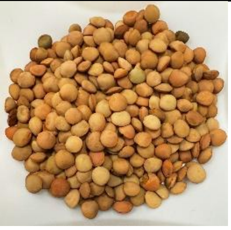 CDC Redwing	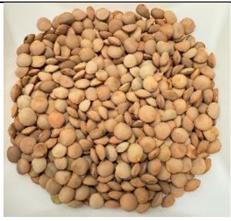 CN 106265
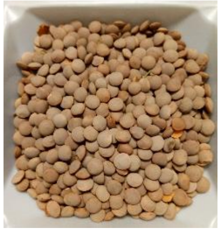 CDC Maxim	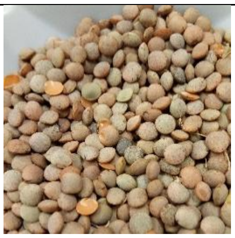 PI 320945 LSP	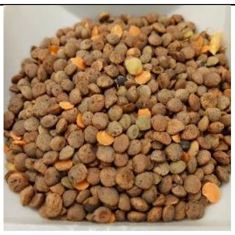 PI 251248 LSP	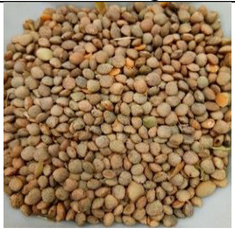 PI 178971	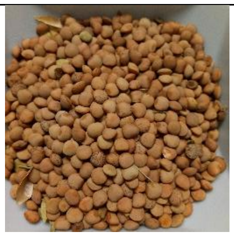 CDC Rosebud
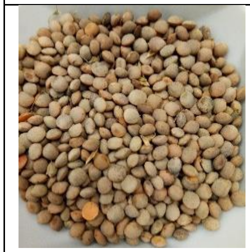 PI 193546				

Green cotyledon lentil	
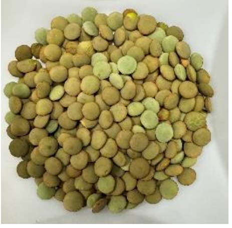 CDC QG-1	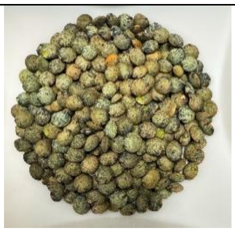 CDC Royale

Yellow cotyledon lentil				
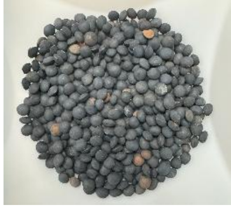 Indianhead	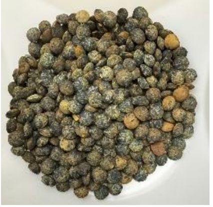 CDC LeMay	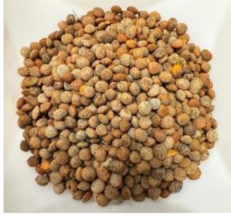 ILL 313	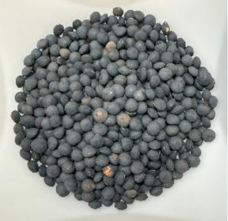 PI 320952 LSP	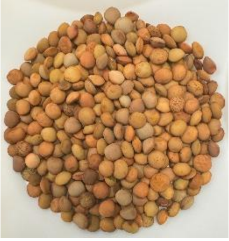 ILL 11548
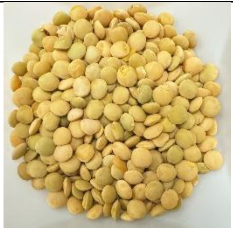 Shasta	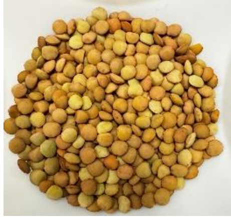 CDC Asterix	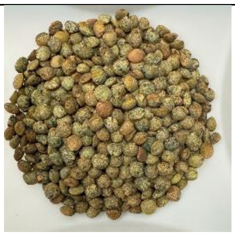 PI 299116 LSP	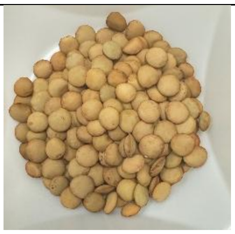 PI 298644 LSP	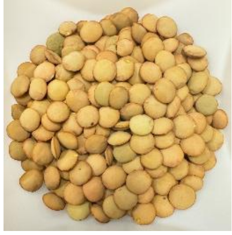 CDC Greenstar
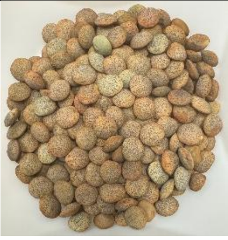 ILL 8072	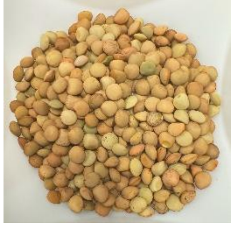 Eston	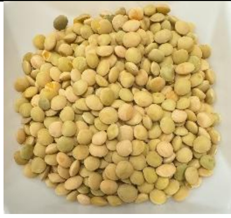 CDC Gold	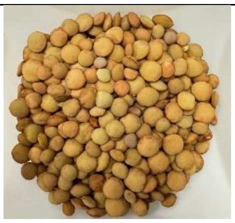 CDC Sedley	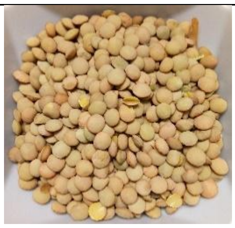 CDC Vantage
